# Recognition of *Aedes aegypti* Mosquito Saliva Protein LTRIN by the Human Receptor LTβR for Controlling the Immune Response

**DOI:** 10.3390/biology13010042

**Published:** 2024-01-12

**Authors:** Su Ning Loh, Ian Russell Anthony, Edem Gavor, Xin Shan Lim, R. Manjunatha Kini, Yu Keung Mok, J. Sivaraman

**Affiliations:** 1 Department of Biological Sciences, National University of Singapore, 14 Science Drive 4, Singapore 117543, Singapore; e0383794@u.nus.edu (S.N.L.);; 2 Department of Pharmacology, Yong Loo Lin School of Medicine, National University of Singapore, Singapore 117600, Singapore

**Keywords:** mosquito, saliva protein, Zika virus, human receptor, EF-hand

## Abstract

**Simple Summary:**

In this study, we present the characterization of *Aedes aegypti* mosquito salivary protein, LTRIN (lymphotoxin β receptor inhibitor), a key facilitator of ZIKV transmission. Injected into the human host during blood-feeding, LTRIN interacts with the human receptor LTβR. We found that LTRIN exists as a thermostable protein with homodimers consisting of an alpha helix-dominant secondary structure with two EF-hand motifs, crucial for withstanding temperature fluctuations during blood-feeding. Despite being an EF-hand protein, its secondary structure remains unaffected by Ca^2+^ binding. ELISA assays and HDX-MS experiments identified the binding region of LTRIN with LTβR. Disrupting the calcium-binding ability in the second EF-hand motif significantly impacted the interaction between LTRIN, particularly the 15 kDa C-terminal protein, ΔLTRIN, and LTβR. Moreover, the decrease in binding affinity of full-length LTRIN suggests the presence of only the truncated form, ΔLTRIN, in the mosquito’s saliva, as the N-terminal region likely covers the interaction site. This insight provides a basis for developing inhibitors against LTRIN as a potential treatment or vaccine target for ZIKV.

**Abstract:**

Salivary proteins from mosquitoes have received significant attention lately due to their potential to develop therapeutic treatments or vaccines for mosquito-borne diseases. Here, we report the characterization of LTRIN (lymphotoxin beta receptor inhibitor), a salivary protein known to enhance the pathogenicity of ZIKV by interrupting the LTβR-initiated NF-κB signaling pathway and, therefore, diminish the immune responses. We demonstrated that the truncated C-terminal LTRIN (ΔLTRIN) is a dimeric protein with a stable alpha helix-dominant secondary structure, which possibly aids in withstanding the temperature fluctuations during blood-feeding events. ΔLTRIN possesses two Ca^2+^ binding EF-hand domains, with the second EF-hand motif playing a more significant role in interacting with LTβR. Additionally, we mapped the primary binding regions of ΔLTRIN on LTβR using hydrogen–deuterium exchange mass spectrometry (HDX-MS) and identified that ^91^QEKAHIAEHMDVPIDTSKMSEQELQFHY^118^ from the N-terminal of ΔLTRIN is the major interacting region. Together, our studies provide insight into the recognition of LTRIN by LTβR. This finding may aid in a future therapeutic and transmission-blocking vaccine development against ZIKV.

## 1. Introduction

Vector-borne diseases are caused by viruses, parasites and bacteria, and they are transmitted through vectors. These vectors can be insects, ticks, mites, fleas or even birds. According to the World Health Organization, vector-borne diseases account for 17% of all infectious diseases, leading to more than 700,000 deaths annually [1,2]. Mosquitoes are known to be the most notorious vectors that spread infectious diseases to the public. Zika virus (ZIKV), dengue virus (DENV), chikungunya virus (CHIKV), yellow fever virus (YFV) and West Nile virus (WNV) are a few notorious viruses that use the mosquitoes as vectors to transmit infectious pathogens to humans through their bites [3,4]. This poses a significant threat to populations worldwide, especially in tropical and subtropical areas where mosquitoes thrive [2,5].

During an infected female mosquito blood-feeding, salivary proteins are injected into the human host along with the viruses. These salivary proteins are known to facilitate the blood-feeding event by impeding blood coagulation, platelet aggregation as well as vasoconstriction [4,6]. Recent studies have shown that some of these salivary proteins not only promote blood-feeding, but also enhance viral infection severity by hampering the human host’s immune responses [4,6,7,8]. For instance, neutrophil-stimulating factor 1 (NeSt1), a 37 kDa protein found in *Aedes aegypti* mosquito saliva, enhances ZIKV pathogenesis by activating neutrophils, which further recruit virus-susceptible macrophages for ZIKV infection at the bite site [9,10].

Among these mosquito-borne diseases, the detection of ZIKV infection is relatively challenging because most infected individuals are asymptomatic. However, some of the ZIKV infected individuals may experience symptoms such as fever, muscle and joint pain, rashes and headaches [11,12]. These symptoms are common in other diseases such as DENV fever and CHIKV making correct identification of ZIKV infected individuals even more difficult. ZIKV is one of the few mosquito-borne diseases associated with neurological complications [13,14]. ZIKV infected adults have a higher risk of developing Guillain–Barré syndrome and radiculopathy [13]. Furthermore, ZIKV can be transmitted vertically from mother to fetus, which can lead to microcephaly and other severe neurological defects in newborns [3,11].

Despite the need for effective therapeutic treatment for ZIKV, no vaccine or drug is currently commercially available [11]. Previous studies have focused extensively on the development of a vaccine or a drug that directly targets the virus particle [15]. However, targeting the virus itself often leads to antibody-dependent enhancement, which in turn, facilitates the infection of other flaviviruses [16]. In recent years, researchers have shifted their focus from pathogen (virus)-centric interventions to vector (mosquito saliva)-centric interventions. The proof-of-concept for the effectiveness and safety of the saliva-centric approach has been demonstrated in the first ever *Anopheles* saliva protein vaccine human trials [17]. Furthermore, passive immunization of mice against mosquito salivary proteins has been shown as a promising alternative strategy for controlling the transmission of ZIKV and other vector-borne diseases [4,10,18].

In this study, we sought to characterize a salivary protein from *Aedes aegypti* named LTRIN (lymphotoxin β receptor inhibitor), which facilitates ZIKV transmission [19]. During a blood-feeding event, LTRIN is injected into the human host and interacts with the receptor LTβR (lymphotoxin β receptor). LTβR has two known binding partners: LTαβ heterotrimer and TNF superfamily member 14 (TNFSF14, also known as LIGHT). Upon binding with the ligand, the dimerization of LTβR is triggered and consequently activates both the canonical and non-canonical NF-κB pathways [19,20]. The activation of the NF-κB signaling pathways results in the synthesis of chemokine and cytokines, promoting the innate immune response [20]. Jin et al. showed that LTRIN binds to the N-terminal domain of LTβR, leading to inhibition of the NF-κB signaling pathway and subsequent production of pro-inflammatory cytokines [16]. Notably, mice that were pre-treated with anti-LTRIN before ZIKV infection survive better than those without treatment, suggesting LTRIN as a promising vaccination target to combat ZIKV infection [19]. 

Here, we report the characterization of ΔLTRIN and its interaction with human LTβR, shedding light on its role in facilitating ZIKV transmission and infection. Through the application of hydrogen deuterium exchange mass spectrometry (HDX-MS) and site-directed mutagenesis, we successfully mapped the interacting interface between ΔLTRIN and LTβR, while also identifying the specific amino acid residues within LTRIN that are involved in these interactions. We additionally examined the secondary structure of LTRIN and assessed the influence of different divalent ions on it.

## 2. Materials and Methods

### 2.1. Maximum Likelihood Phylogenetic Tree

Similar protein sequences of LTRIN (XP_011493643.1) and MCFD2 (CAD38756.1) were identified using the Protein BLAST tool [21]. Only proteins having fasta sequences with more than 70% similarity to *Insecta* and *Hominidae* were selected and combined for analysis. Sequence alignment and generation of maximum likelihood phylogenetic tree were performed using the program MEGA11 (Molecular Evolutionary Genetics Analysis version 11) with bootstrap value of 1000 [22].

### 2.2. Protein Expression and Purification

The full length LTRIN consists of a signal peptide and highly disordered region at its N-terminal. Only the residue from 86 to 210 was codon-optimized and cloned into a modified pET28a vector with an N-terminal 6-His tag followed by thrombin cleavage site. The vector was introduced into *E.coli* BL21 strain and was grown in LB media with 100 μg/mL of Amp^+^ until the OD_600_ reached 0.8. Then, 250 μM of isopropyl β-d-1-thiogalactopyranoside (IPTG) was added to the bacterial cell culture to induce the expression of ΔLTRIN, and the culture was allowed to continue to grow at 18 °C for 16 to 20 h.

The cell culture was pelleted at 3500 rpm for 30 min using a Beckman Coulter Centrifuge JLA-8.1 Rotor (Beckman Coulter, Brea, CA, USA). The purification procedures are described elsewhere [19]. Briefly, the pellets were washed once with PBS then resuspended in lysis buffer (50 mM Bis-Tris, 300 mM NaCl, 5% glycerol, 2 mM DTT pH 6.5). The cell was lysed using sonicator in the presence of 100 mM PMSF, cOmplete™ EDTA-free protease inhibitor cocktail (Thermo Fisher Scientific, Waltham, MA, USA) and 0.5% of Triton X100. The lysed product was centrifuged at 18,000 rpm using Beckmann Coulter Centrifuge JA-20 Rotor ((Beckman Coulter, Brea, CA, USA) for 30 min at 4 °C. The supernatant was subjected to Ni-NTA affinity chromatography followed by anion exchange chromatography to remove impurities. The His-tag was cleaved by incubating with thrombin at 4 °C overnight then further purified via size exclusion chromatography by using Superdex 75 column (GE HealthCare, Chicago, IL, USA). ΔLTRIN was kept in 50 mM Bis-Tris pH 6.5, 100 mM NaCl, 2% glycerol. The other mutants and constructs were purified using the same methods as the WT ΔLTRIN.

The extracellular domain of LTβR (residues Gln 31-Met 227) was expressed in Sf9 cell using Bac-to-Bac system according to manufacturer’s recommendation (Invitrogen, Watham, MA, USA). Briefly, the extracellular domain was first cloned into pFastBac HTB vector with gp67 signal peptide and 6-His tag addressed at the N-terminal. The sequence confirmed plasmid was transformed into DH10EmBacY competent cells and selected using blue-white screening. The recombinant bacmid DNA was extracted and transfected into Sf9 cells with CellFectin II transfection reagent (Invitrogen, Waltham, MA, USA). About 5–7 days following transfection, when cells exhibited visible cytopathic effect (>70%), the virus-containing supernatant was harvested as V_0_ through centrifugation at 1000× *g* for 20 min. The V_0_ baculovirus was subsequently utilized to infect Sf9 in order to produce V_1_ as the baculovirus stock. High Five™ cells at a density of 2.0 × 10^6^ cells/mL were infected by V_1_ LTβR baculovirus at a concentration of 2–4% *v*/*v* for protein production. The infected cells were harvested at 60 h post-infection. The medium that contained secreted LTβR was subjected to Ni-NTA affinity chromatography and further purified using methods mentioned above. Similar method was used to prepare N40G N177G LTβR to remove N-linked glycosylation. Both LTβR and N40G N177G LTβR were stored in 50 mM Tris-HCl pH 7.5, 100 mM NaCl and 2% glycerol buffer.

### 2.3. Protein Refolding of LTβR

The extracellular domain of LTβR was cloned into a modified pET28a vector with 6-His tag and HRV 3C Protease cleavage site at the N-terminal. The protein was refolded following standard protocol described elsewhere [23]. Briefly, the cells were sonicated in 50 mM Tris-HCl pH 8.0, 300 mM NaCl, 2 M urea and 2% glycerol. The inclusion body was dissolved in 100 mM Tris-HCl, 8 M urea pH 8.0, then further purified using Ni-NTA column. The purified denatured protein sample was added dropwise into an agitating refolding buffer (100 mM Tris HCl, 2 mM EDTA, 400 mM L-arginine, 0.5 mM oxidized glutathione, 5 mM reduced glutathione, pH 8.0). The refolded protein was concentrated using Vivaspin 20 centrifugal concentrator (Cytiva, Marlborough, MA, USA) with a molecular weight cutoff at 10 kDa. The concentrated protein was buffer exchanged to 50 mM Tris-HCl pH 7.5, 100 mM NaCl and 2% glycerol by using Superdex 75 column (GE HealthCare, Chicago, IL, USA). 

### 2.4. Sedimentation Velocity-Analytical Ultracentrifugation of ΔLTRIN

The SV-AUC was carried out for ΔLTRIN in 25 mM Tris HCl, 50 mM NaCl pH 7.5. The samples were examined by Beckman ProteomeLab XL-1 analytical ultracentrifuge (Beckman Coulter, Brea, CA, USA) equipped with an-60 Ti rotor. Next, 400 μL of protein sample (0.5 mg/mL) was loaded into epon double-sector centerpieces equipped with quartz windows and centrifuged at 40,000 rpm, 20 °C in vacuum until full sedimentation. Then, 70 readings were obtained at A280 nm, with a 6-min interval between each reading. The scans from the 5th to the 50th scan were analyzed using SEDFIT16-1c software, employing the “Continuous c(s) distribution” model.

### 2.5. Dynamic Light Scattering of ΔLTRIN

The DLS measurements were performed to evaluate the homogeneity of ΔLTRIN across different concentrations [24]. The DLS instrument was operated according to the manufacturer’s guidelines. Briefly, the ΔLTRIN samples of different concentrations were centrifuged at 15,000 rpm for 10 min before measurement and then 4 µL of the sample was added to the cuvette to perform measurement. All the scattered light intensity was measured using DynaPro Nanostar (Wyatt Technology, Santa Barbara, CA, USA).

### 2.6. Ruthenium Red Assay of ΔLTRIN

With BSA as negative control and MCFD2 as positive control, the purified ΔLTRIN was subjected to electrophoresis on 15% SDS-PAGE gel. A total of 2 μg of each protein sample was loaded into every well. The gel was then transferred onto PVDF membrane via Trans-Blot^®^ Turbo™ Transfer System (Bio-Rad, Hercules, CA, USA). The membrane was then washed with washing buffer (10 mM Tris-HCl, 60 mM KCl, 5 mM MgCl_2_, pH 7.5) 3 times and then stained with ruthenium red staining solution for 20 min at room temperature. The ruthenium red staining solution was prepared by dissolving ruthenium red in the washing buffer to a final working concentration of 25 mg/L [25]. 

### 2.7. Isothermal Titration Calorimetry of ΔLTRIN with Different Divalent Ions

The ITC measurements were conducted using MicroCal PEAQ-ITC (Malvern Panalytical, Malvern, UK). The protein samples were buffer exchanged to 50 mM Bis-Tris pH 6.5, 100 mM NaCl and concentrated to a series of concentrations ranging from 100 µM to 300 µM. The same buffer was used for preparing the ligands (CaCl_2_, MgCl_2_, ZnCl_2_) up to 5 mM. The measurement was carried out at 25 °C, with stirring speed at 500 rpm. Each injection was performed with a 2 µL and 150 s spacing between injections. The data were analyzed using MicroCal PEAQ-ITC analysis software (version 1.40) with one set of sites fitting the model.

### 2.8. Gel Mobility Shift Assay of ΔLTRIN

The procedure for conducting the gel mobility assay was performed following the method described elsewhere [25]. Briefly, we prepared 15% SDS gel with 3 mM Ca^2+^ and 3 mM EDTA. Next, we prepared an equal amount of BSA (negative control), MCFD2 (positive control) and ΔLTRIN in sample loading buffer, then loaded equal volume of all samples to both the SDS gel with 3 mM Ca^2+^ and 3 mM EDTA. The final quantity of 2 μg of each protein sample was added to every well. The gel was visualized by Coomassie blue staining solution. 

### 2.9. Circular Dichroism of ΔLTRIN and its Mutants

Around 0.3 mg/mL of ΔLTRIN, D123A, D180A and D123A D180A mutants were prepared in 5 mM Tris-HCl, 25 mM NaCl pH 7.5 buffer. The samples were centrifuged at 15,000 rpm, 4 °C before loading into the cuvette. Subsequently, 200 μL of centrifuged protein sample with final concentration of 2 mM of EGTA or 5 mM of CaCl_2_ was loaded into the cuvette. Washes were conducted after each measurement. The far UV Circular Dichroism (FUV-CD) spectroscopy was measured using J-1100 Circular Dichroism spectrometer (Jasco Inc., Tokyo, Japan). The CD spectra profile of ΔLTRIN as a function of temperature was also obtained by measuring at 222 nm across 20 to 100 °C.

### 2.10. ELISA

The ELISA assay was conducted according to Themo Fisher Scientific’s (Waltham, MA, USA) protocol. Briefly, 10 ug of ΔLTRIN or FL LTRIN or ^119^LTRIN^210^ was coated on each well of 96-well flat bottom plate overnight at 4 °C. After incubating with 5% of BSA blocking buffer in PBST for 2 h at 37 °C, 0.15 µg of WT LTβR, refold LTβR and LTβR N40G N177G were added to each well and incubated at room temperature for another 2 h. Rabbit monoclonal against LTΒR (80178-T16, Sino Biological, Beijing, China) and anti-rabbit IgG conjugated with HRP (A120-101P, Bethyl Laboratories, Montgomery, AL, USA) were used as primary and secondary antibodies, respectively. TMB-ELISA substrate (3,3’,5,5’-Tetramethylbenzidine) was added for color development and was measured at 450 nM using plate reader (Infinite^®^ M Nano+, Tecan, Männedorf, Switzerland). 

### 2.11. Hydrogen-Deuterium Exchange Mass Spectrometry

In this experiment, 90 pmol of ΔLTRIN and LTβR proteins was used for each HDX experiment (apo ΔLTRIN, or ΔLTRIN and LTβR complexes) from stock solution containing 1 mg/mL ΔLTRIN and 2 mg/mL LTβR.

Deuterium exchange buffer was prepared by drying aqueous PBS buffer pH 7.4 to remove all H_2_O, and 99.9% D_2_O was added to reconstitute to form the deuterium exchange buffer. Amide hydrogen deuterium exchange reaction was initiated by adding apo ΔLTRIN, or ΔLTRIN and LTβR complexes into 10× volume of deuterium exchange buffer to yield a final D_2_O concentration of 89.9%. Deuterium labelling was then carried out at 2 different deuterium exposure times of 1 min and 100 min.

HDX reaction was quenched by reducing pH of the reaction to 2.5 by adding formic acid in GnHCl to achieve a final concentration of 1.5 M GnHCl. Quenching of reaction was performed for 1 min on ice to further slowdown any back exchange reaction. All deuterium exchange reactions were performed in triplicate and the final deuterons uptake value for each peptic digested fragment reported is an average value of the triplicates without back exchange correction.

All quenched samples were injected into nano-UPLC HDX sample manager (Waters, Milford, MA, USA), online pepsin digestion was performed using a Waters Enzymate BEH pepsin (2.1 × 30 mm) column in 0.05% formic acid in water at 100 mL/min. Proteolyzed pepsin fragment peptides were trapped by a 2.1 × 5 mm C18 trap (ACQUITY BEH C18 VanGuard Pre-column, 1.7 mm, Waters, Milford, MA, USA) and eluted with an 8–40% gradient of acetonitrile in 0.1% formic acid at 40 mL/min into a reverse phase column (ACQUITY UPLC BEH C18 Column, 1.0 × 100 mm, 1.7 mm, Waters) by nano ACQUITY Binary Solvent Manager (Waters, Milford, MA, USA). Peptides were ionized by electrospray into SYNAPT G2-Si mass spectrometer (Waters, Milford, MA, USA) acquiring in MSE mode for detection and mass measurements. Next, 200 fmol/mL of [Glu1]-fibrinopeptide B ([Glu1]-Fib) was simultaneously injected into the mass spectrometer at a flow rate of 10 mL/min for continuous calibration during sample acquisition [26].

All deuterium uptake calculations were performed using DYNAMX 3.0 software (Waters, Milford, MA, USA). Deuteron uptake per peptide was obtained by subtracting mass centroid of deuterium exposed peptides (1 min and 100 min) with the mass centroid of the undeuterated peptide. Differences between ΔLTRIN and LTβR complex with ΔLTRIN apo state were then obtained by subtracting deuterons uptake of all peptides in the ΔLTRIN and LTβR complex with ΔLTRIN apo state and represented as a difference plot of peptides spanning from N to C terminus. 

## 3. Results

### 3.1. Evolutionary Relationship of LTRIN with Insecta and Hominoidea

**Figure 1:**
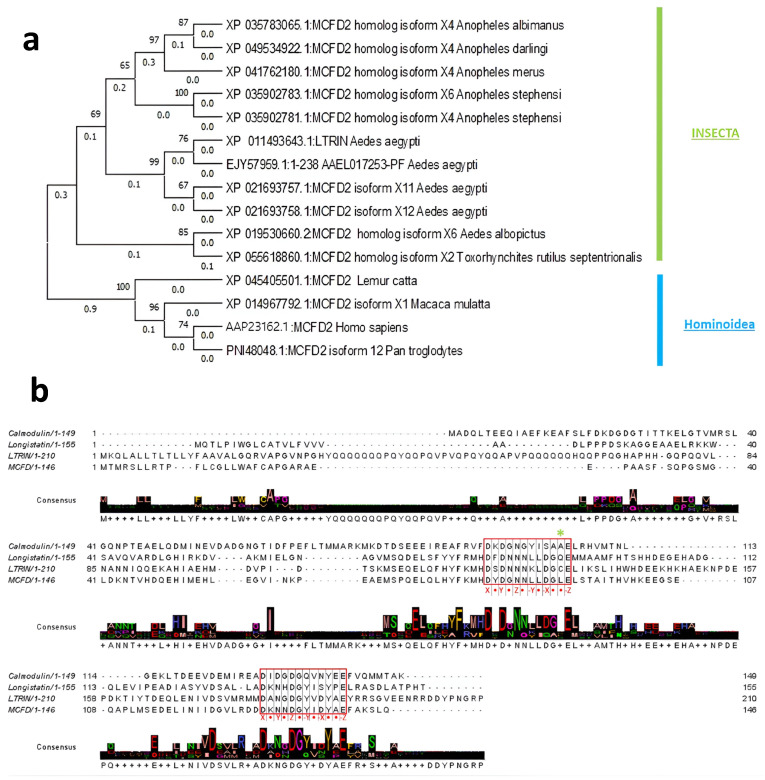
(**a**) Maximum likelihood phylogenetic tree of mosquito salivary protein LTRIN from *Aedes Aegypti* (XP_011493643.1) and multiple coagulation factor deficiency protein 2 (MCFD2) from insect and human. (**b**) Sequence alignment of LTRIN with human MCFD2 and calmodulin. The EF-hand motif is highlighted using a red box. The Ca^2+^ binding loop sequence is highly conserved among all three proteins, arranging in X • Y • Z • -Y• -X • • -Z fashion, where X and -Z are usually occupied by Asp and Glu. The C133 position of LTRIN is denoted with *.

Maximum likelihood phylogenetic tree was created to illustrate a hypothesized evolutionary path of mosquito salivary protein LTRIN from *Aedes aegypti* (XP_011493643.1) with closely related proteins. Through a BLASTp analysis, we observed that multiple coagulation factor deficiency protein 2 (MCFD2) of *Aedes aegypti* (XP_021693757.1), *Aedes albopictus* (XP_019530660.2) and *Anopheles stephensi* (XP_035902781.1) *Insecta* share a sequence identity of 99.05%, 67.18% and 77.78% to LTRIN, respectively. The main function of MCFD2 in *Homo sapiens* is to shuttle various metabolic proteins between the endoplasmic reticulum (ER) and the ER-Golgi intermediate compartment during blood clotting [27]. LTRIN of *Aedes aegypti* and MCFD2 of *Homo sapiens* (AAP23162.1) share a 41.94% sequence identity, and is mainly contributed by the presence of EF-hand motif. Further examination of the evolutionary relationship of these homolog proteins was performed together with these proteins’ respective superfamilies. Thus, MCFD2 protein of *Homo sapiens* was also subjected to a BLASTp. Results from both BLASTp were collated and aligned to create a maximum likelihood phylogenetic tree (Figure 1a). The phylogenetic tree shows a good confidence value as all bootstrap values are over 60% and six out of the eleven values are over 90%. *Insecta* and *Hominoidea* distinctively split into brunches with lengths of 0.3 and 1.0, respectively, which reveal greater genetic and functional change in *Hominoidea* than *Insecta* when compared with the ancestral state. LTRIN has a shorter brunch length of 0.5, as compared to the 1.0 of *Homo sapiens* MCFD2. In humans, MCFD2 is involved in transport of FV and FVIII from ER to Golgi [27]. To understand the mode of recognition of receptor LTβR and LIRIN, in the following sections we present the characterization of LTRIN and its interactions with the receptor. 

### 3.2. LTRIN Exists as a Dimer

**Figure 2:**
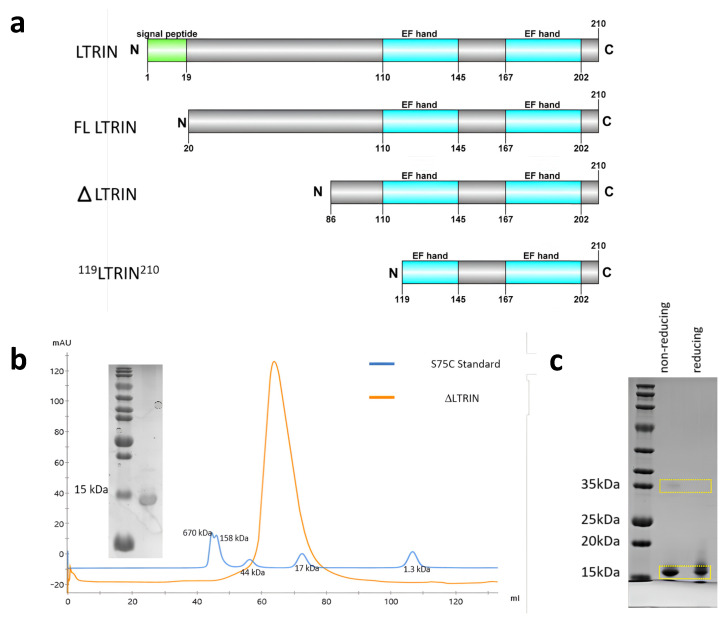
(**a**) The full length of LTRIN consists of signal peptide and two calcium-binding EF-hand domains. (**b**) ΔLTRIN size exclusion chromatography elution profile after Ni-NTA affinity column and ion exchanger chromatography. The blue line represents the standard curve, indicating that ΔLTRIN (orange) eluted between 44 kDa and 17 kDa, which implies the formation of a dimer. (**c**) SDS PAGE with and without reducing agent (β-mercaptoethanol) was conducted to analyze the dimerization of ΔLTRIN. In the presence of the reducing agent, most of ΔLTRIN was observed in its monomeric state, indicating that disulfide bond formation is not the main factor contributing to ΔLTRIN’s dimerization. ΔLTRIN is highlighted with a yellow box.

The full-length LTRIN is 22 kDa with a signal peptide and a highly disordered region at the N terminal. It was reported that the 15 kDa C-terminal region of LTRIN alone is sufficient to interact with LTβR [19]. Hence, the 15 kDa truncated C-terminal ^86^LTRIN^210^ (hereafter referred as ΔLTRIN) is expressed in *E. coli* bacterial cells (Figure 2a). The protein is purified using size exclusion chromatography after passing through Ni-NTA affinity chromatography. The elution profile shows that ΔLTRIN is eluted as a dimer with a molecular weight of around 30 kDa (Figure 2b). The dimerization of ΔLTRIN is further confirmed using sedimentation velocity-analytical ultracentrifugation (SV-AUC) sedimentation velocity (Supplementary Figure S1a). The ΔLTRIN has a size distribution at 30.6 kDa, supporting its dimerization. 

Occasionally, protein oligomerization is triggered by high protein concentrations or interactions between proteins and ions [28,29,30]. To elucidate whether the oligomerization state of ΔLTRIN is driven by concentration or not, dynamic light scattering (DLS), as well as static light scattering (SLS) of different concentrations of ΔLTRIN, ranging from 1 mg/mL to 20 mg/mL, were measured. Supplementary Figure S1b shows that the dimerization of ΔLTRIN remains relatively stable across the various concentrations measured, as evidenced by the consistent and narrow peak in the DLS profile. Similarly, there was no effect on the dimerization state of ΔLTRIN in the presence of Ca^2+^ or EDTA, indicating that interactions with ions do not play a role in ΔLTRIN dimerization (Supplementary Figure S1c,d).

The sulfhydryl group or thiol group (S-H) of cysteine can form a disulfide bond (S-S) with another cysteine residue. These bonds are known to play a crucial role in protein folding and stabilization as well as cysteine-mediated oligomerization [31,32]. ΔLTRIN consists of a cysteine residue located at position 133, which can potentially form a disulfide bond with the cysteine residue from another molecule, and could form the dimers. SDS-PAGE was conducted with and without the addition of reducing agent (β-mercaptoethanol). In the absence of the reducing agent, a small portion appeared in dimer form (band around 35 kDa), while most of the ΔLTRIN was observed in its monomeric state (band at 15 kDa) (Figure 2c), suggesting that disulfide bond formation is not the primary cause of ΔLTRIN dimerization. We further introduce a point mutation at position 133 to make C133Q/L variant taking clues for the sequence alignment with its insect homolog longistatin and human homolog MCFD2 (Figure 1b) [33]. The size exclusion chromatography elution profile of C133Q/L indicates that it still forms a dimer and is further confirmed by DLS (Supplementary Figure S1e,f). This suggests that the dimerization is not mediated by the disulfide bonds, but it could be facilitated by electrostatic interactions involving the charged residues found in LTRIN [34].

### 3.3. LTRIN Has EF-Hand Motifs and Binds to Ca^2+^ and Other Divalent Ions

The EF-hand motif is characterized by a helix-loop-helix secondary structure with two α-helices connected by a Ca^2+^ binding loop. This binding loop consists of 12 amino acids and is typically structured in the fashion of X • Y • Z • -Y• -X • • -Z, where X, Y, Z, -Y, -X, -Z denote the amino acids that coordinate with Ca^2+^ ions and • represents the intervening residues. The X and -Z positions are highly conserved, and are usually occupied by Asp and Glu, respectively. Asp or Asn is commonly found at position Y, whereas positions Z and -X can be occupied by either Asp, Asn, or Ser. The -Y position is rather flexible; many different residues have been observed [35,36,37,38]. Examining the protein sequence of LTRIN, we observed two sets of potential EF-hand motifs with the Ca^2+^ binding loop sequence as follows: ^123^DSDNNNKLDGCE^134^ and ^180^DANGDGYVDYAE^191^. These loop sequences are highly negatively charged with Asp and Glu at position X and -Z, respectively, and these sequences are highly conserved among other EF-hand domain proteins (Figure 1b). 

Ruthenium red is a polycationic stain that has been used to identify proteins with EF-hand motif [25,39]. We performed ruthenium red staining using MCFD2 as a positive control. We found that ΔLTRIN binds to ruthenium red and confirmed the presence of EF-hand motif (Supplementary Figure S2a). Further, the Ca^2+^ binding ability of ΔLTRIN is confirmed by isothermal titration calorimetry (ITC) with K_D_ value 27.3 × 10^−6^ ± 9.43 × 10^−6^ M (Supplementary Figure S2c). The Ca^2+^ binding EF-hand motif is also known to accommodate other divalent ions such as Mg^2+^ and Zn^2+^ [40,41]. We also performed ITC experiments with these two ions. Interestingly, ΔLTRIN does not interact with Mg^2+^ and has lower binding affinity with Zn^2+^ compared to Ca^2+^ (Supplementary Figure S2c).

In some cases, the binding of Ca^2+^ to EF-hand motif can lead to significant conformational changes. In order to examine the possible conformational changes of ΔLTRIN upon binding with Ca^2+^, gel mobility shift assay has been carried out [25]. Supplementary Figure S2b shows that the presence of Ca^2+^ did not significantly alter the mobility of ΔLTRIN, indicating that binding with Ca^2+^ does not result in a significant conformational change in it. This conclusion is also supported by Circular Dichroism (CD) studies (discussed below).

### 3.4. LTRIN Has a Stable Alpha-Helix Dominant Structure over a Wide Range of Temperatures

**Figure 3:**
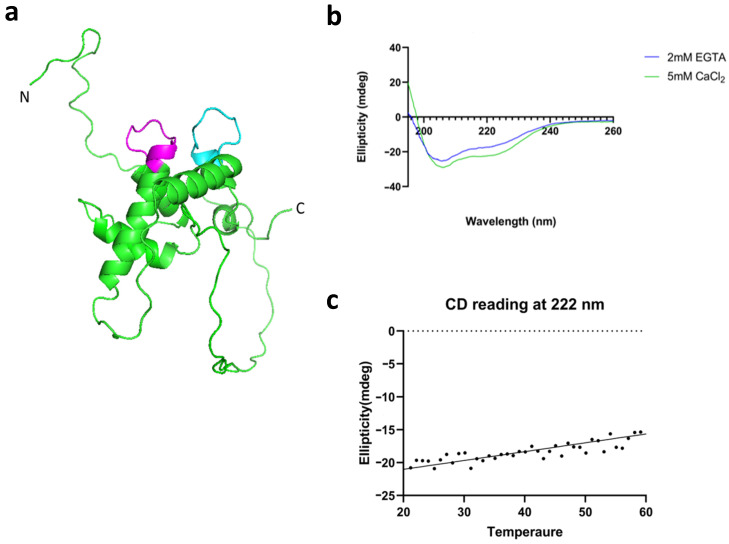
(**a**) The full length LTRIN structure modelled using AlphaFold. The N-terminal is showing random coiling. The Ca^2+^ binding loops are highlighted in magenta (the first binding loop) and cyan (the second binding loop). (**b**) Circular dichroism (CD) spectra of ΔLTRIN in presence of CaCl_2_ and EGTA. ΔLTRIN preserved its α-helix dominant secondary structure with or without the presence of Ca^2+^. (**c**) CD spectra reading of ΔLTRIN at 222 nm from 20 °C to 60 °C showing that ΔLTRIN has high thermostability.

The secondary structure of ΔLTRIN was analyzed using CD (Figure 3b and Figure S1g). The results showed two negative peaks detected at approximately 222 nm and 208 nm, implying that ΔLTRIN has an α-helix dominant secondary structure, and such observations are consistent with the predicted secondary structure (Figure 3a and Figure S1h) [42]. Further, the CD spectra of ΔLTRIN were used to estimate its secondary structures through three different methods (SELCON3, CONTINLL and CDSSTR), and subsequently compared with the structure predicted by AlphaFold [43]. Both experimental and computational analyses were consistent, which showed that ΔLTRIN possesses mainly alpha-helix and loop structures (Supplementary Table S1).

In contrast to MCFD2, ΔLTRIN possesses a well folded secondary structure even in the absence of Ca^2+^. Regardless, adding Ca^2+^ did not alter the overall secondary structure of ΔLTRIN; instead, it undergoes more subtle structural changes as evidenced by the similar but deeper spectral patterns observed in both the absence and presence of Ca^2+^ (Figure 3b). This observation is consistent with the gel mobility assay results (Supplementary Figure S2b). 

Thermostability is an important feature of protein to maintain its secondary structure and perform its activity effectively. We investigated the conformational changes of ΔLTRIN at various temperatures. The CD data show that ΔLTRIN has a relatively stable secondary structure across a wide range of temperatures. Even heating up to 100 °C, ΔLTRIN did not completely lose its secondary structure; instead, it only underwent partial denaturation and indicated that LTRIN is highly thermostable (Figure 3c,d).

### 3.5. Disulfide Bonds in LTβR 

**Figure 4:**
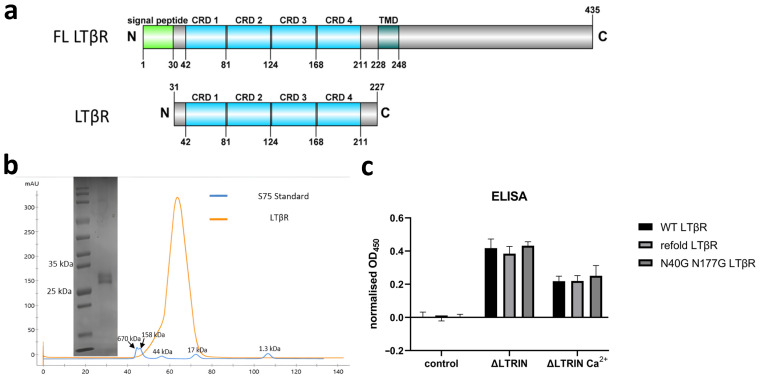
(**a**) Full length LTβR is a transmembrane protein with signal peptide at N-terminal. The extracellular domain is used for the study. (**b**) The cysteine rich extracellular domain (CRD) of LTβR expressed in insect High Five™ cell line via baculovirus expression system. The blue line indicates the standard curve, demonstrating that LTβR (orange) possesses a molecular weight ranging from 44 kDa to 17 kDa. (**c**) ΔLTRIN interacts with LTβR and the interaction is not dependent on either divalent ions or glycosylation. TMD: transmembrane domain.

LTβR, a type-I transmembrane glycoprotein, belongs to the tumor necrosis factor (TNF) superfamily. Its extracellular domain consists of four cysteine-rich domains (CRD1–CRD4), which are responsible for the recognition of other TNF ligands. Each domain is approximately 40–44 amino acid residues in length. CRD1–3 consists of six Cys residues, forming three intradomain disulfide bonds within each domain, while CRD4 has four Cys residues, forming two disulfide bonds (Cys170–Cys185 and Cys191–Cys210). N-linked glycosylation sites are present at positions Asn40 and Asn177 of LTβR [20,44]. We have expressed this protein using baculovirus expression system, and it is secreted with glycosylation (Figure 4b).

### 3.6. The Interactions between LTRIN and LTβR Are Not Affected by the Glycosylation of LTβR or Divalent Ions

ELISA binding experiments were conducted to investigate the binding between ΔLTRIN and LTβR. Our experiments showed that the binding between ΔLTRIN and extracellular domain of LTβR was independent of the presence of divalent Ca^2+^ ions (Figure 4c). It is well known that the metal ion Zn^2+^ is widely used to precipitate proteins [45]. In our case, the excessive amount of Zn^2+^ also caused ΔLTRIN precipitation and it could explain the negative value in ELISA binding (Supplementary Figure S2d). Although ΔLTRIN consists of canonical EF-hand motif that binds to Ca^2+^, our results showed that it does not require the divalent ions to interact with LTβR. In fact, the binding is slightly impeded in the presence of metal ion (Figure 4c). 

Glycosylation is known to play an important role in regulating cell functions including intercellular communication and intracellular signaling pathways [46,47,48]. In order to examine the effect of LTβR glycan in ΔLTRIN interactions, point mutations at these two glycosylation sites (N40G N177G LTβR) were generated. The data show that N40G N177G LTβR has similar binding affinity toward ΔLTRIN compared to WT LTβR, indicating that the interaction between LTβR and LTRIN is independent of the glycosylation of LTβR (Figure 4c). 

### 3.7. Mapping the Interaction Sites of ΔLTRIN with LTβR 

**Figure 5:**
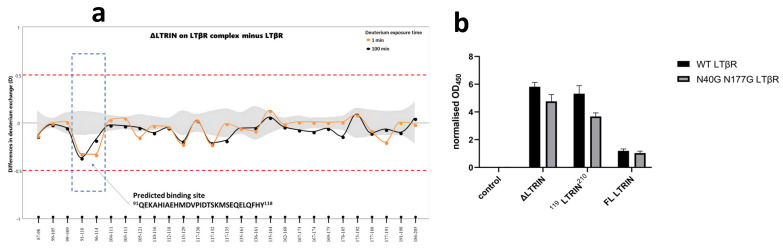
The interacting site of ΔLTRIN on LTβR. (**a**) Plot depicting differences in deuterium exchange (Y-axis) between apo and bound form of ΔLTRIN. The interacting peptide sequence is proposed to be ^91^QEKAHIAEHMDVPIDTSKMSEQELQFHY^118^. (**b**) The ELISA shows both FL LTRIN and truncated ^119^LTRIN^210^ in binding.

HDX-MS is performed to determine the interaction site of ΔLTRIN with LTβR. Interestingly, the HDX-MS result revealed that the N-terminal of LTRIN (^91^QEKAHIAEHMDVPIDTSKMSEQELQFHY^118^) is playing the key role in interacting with LTβR (Figure 5a). 

According to the predicted structure using both AlphaFold and PSIPRED, the N-terminal of LTRIN including the proposed interacting region displays as random coil structure followed by a helix–loop–short helix–loop structure before entering the E-helix of the first EF-hand motif (Figure 3a and Figure S1h). The HDX-MS data suggested that this helix–loop–short helix–loop structure together with the N-terminal of the E-helix of the first EF-hand are responsible for the interaction with LTβR. It would be intriguing to investigate the interaction between full-length LTRIN and LTβR, as well as determine whether the random coil N-terminal region plays a role in this interaction within the context of the full-length protein. To validate the involvement of the N-terminal interacting site, we expressed another two constructs termed ^119^LTRIN^210^ by deleting the interacting segment suggested by HDX-MS data and also the full length of LTRIN (hereafter referred as FL LTRIN without the signal peptide).

ELISA was carried out using both the FL LTRIN and the construct ^119^LTRIN^210^ (Figure 5b). The binding affinity between the FL LTRIN and LTβR is significantly reduced while that of ^119^LTRIN^210^ shows a slight reduction in binding when compared to the ΔLTRIN.

### 3.8. The Second EF-Hand of ΔLTRIN Plays an Important Role in Interacting with LTβR 

**Figure 6:**
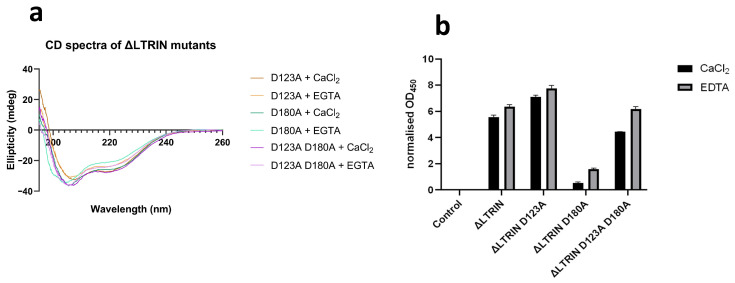
Altering the calcium-binding loop of EF-hands in ΔLTRIN. (**a**) The secondary structure of ΔLTRIN remains unchanged regardless of the ability to bind calcium or the presence of Ca^2+^. (**b**) The ELISA results indicate that mutating D123A enhances interaction, while mutating D180A disrupts the interaction between ΔLTRIN and LTβR.

Despite the fact that the presence of Ca^2+^ does not affect the secondary structure of ΔLTRIN, modifications were implemented on its EF-hands to assess how its interaction with LTβR would be affected by its binding ability with calcium. D123A, D180A and D123A D180A mutants were made to disrupt the calcium-binding ability of either the first or second or both EF-hands in ΔLTRIN. The secondary structures of all the mutants were examined using CD and compared to wild-type ΔLTRIN. Figure 6a shows that the α helix-dominated secondary structure of ΔLTRIN was well preserved in all the mutants in the presence of either Ca^2+^ or EDTA, further indicating that disruption of calcium binding in the EF-hands did not affect the overall secondary structure of ΔLTRIN.

ELISA was conducted on all the mutants, and it was observed from the results that the D123A mutation, which impaired the calcium-binding capability of the first EF-hand motif, did not decrease but rather slightly enhanced the interaction between ΔLTRIN and LTβR. However, the D180A mutant, which disrupted the calcium-binding ability of the second EF-hand motif in ΔLTRIN, exhibited a significant reduction in ΔLTRIN interaction with LTβR. This suggests that the second EF-hand of ΔLTRIN plays an important role in interacting with LTβR. Notably, the interaction between ΔLTRIN and LTβR was not completely abolished by the double mutant D123A D180A. In fact, the interaction was restored to a level similar to that of the wild-type ΔLTRIN, suggesting the D123A mutation favors the interaction (Figure 6b). 

These findings indicate that calcium-binding capability of both EF-hands contributes to the interaction between ΔLTRIN and LTβR, with the second EF-hand motif playing a more crucial role. 

## 4. Discussion

The maximum likelihood phylogenetic tree shows that LTRIN and MCFD2 share the same origin. We assessed the protein sequence and the predicted secondary structure of both LTRIN and MCFD2 and showed that they are highly similar. Therefore, we speculate that MCFD2 will likely interact with LTβR due to its high sequence similarity and the presence of EF-hand motif. As *Hominoidea* show a greater genetic and functional change than *Insecta* when compared to the ancestral state, LTRIN has closer relations to the ancestral state than *Homo sapiens* MCFD2, which might be the reason for LTβRs’ preferential binding of LTRIN.

EF-hands motif is known to bind with Ca^2+^ and sometimes the binding will give rise to conformational change [49,50,51]. MCFD2 has a random coiled secondary structure as an apo protein. Its C-terminal undergoes conformational changes to form the canonical helix-loop-helix structure upon interaction with Ca^2+^ [52,53]. Although the majority of EF-hand proteins undergo conformational changes upon binding with Ca^2+^, some proteins maintain the same secondary structure even in the presence of Ca^2+^ [54,55]. As suggested by our data, ΔLTRIN holds a well folded conformation in apo form and the presence of Ca^2+^ ion has limited effect on its secondary structure. This is further supported by the observation that eliminating the ability of EF-hand motifs of ΔLTRIN to bind calcium ions did not cause significant alterations in its secondary structure. 

Our data shows that ΔLTRIN forms a homodimer regardless of the protein concentration or the presence of Ca^2+^, and this is not contributed through the formation of disulfide bond. We suggest that the dimerization is through hydrophobic and electrostatic interaction between the EF-hand motifs of ΔLTRIN [56]. This implies that the Ca^2+^-binding domain can also serve as a dimerization unit. In fact, a few of the EF-hand Ca^2+^-binding proteins are reported to form dimers through hydrophobic interactions between the EF-hand motifs [57]. 

Oligomerization in EF-hand domain proteins is commonly observed as it provides extra interacting sites. Activation of the NF-κB signaling pathway is through dimerization of LTβR. Jin et al., reported that LTRIN disrupts the dimerization of LTβR; hence, diminishing the signaling pathway [19]. We speculate that homodimer of ΔLTRIN will interact with two adjacent LTβR forming a heterotetramer, resulting in the prevention of LTβR dimerization.

Blood-taking is a very stressful event for a mosquito as it requires dealing with abrupt temperature changes, from 22 to 37 °C [58]. Similarly, the salivary protein that is injected into the human host needs to have high thermostability to withstand the host body temperature and maintain its function. The temperature-dependent CD shows that LTRIN can preserve its structure regardless of the temperature.

According to the ELISA result, the binding affinity of LTβR, N40G, N177G LTβR and refolded LTβR with ΔLTRIN suggests that the interaction is not dependent on post translational modifications of LTβR. Interestingly, the presence of Ca^2+^ ions slightly disrupt the interaction. We hypothesize that this could be due to the subtle conformational change in ΔLTRIN induced by the ions. As indicated by the CD data, the interaction of ΔLTRIN with Ca^2+^ ion leads to a more compact and well-folded structure, leading to decreased flexibility and subsequently reduced binding affinity.

From the result of HDX-MS, it was identified that the N-terminal region of ΔLTRIN is responsible for interacting with LTβR. However, the FL LTRIN which composes the interacting site showed a great reduction in binding with LTβR. The N-terminal region of the full-length LTRIN is predicted to be a 65-amino acid-long segment that lacks defined structure and adopts a random coil conformation. We hypothesized that this flexible random coil may adopt certain conformations which hinder its ability to interact with LTβR, resulting in reduced binding affinity. This also explains why only the ΔLTRIN, rather than the intact full-length protein, is found in mosquitoes’ saliva [19]. The randomly coiled portion needs to be removed through cleavage in order for the interaction site to become exposed and allow binding with LTβR, and subsequently, inhibit the NF-κB signaling pathway. Future studies are necessary to validate the role of the N-terminal region of FL LTRIN in mediating the interaction with LTβR.

The removal of the interacting site from ^119^LTRIN^210^ did not completely abolish its binding with LTβR but only resulted in a slight decrease. This observation can be explained by two hypotheses. Firstly, the construct ^119^LTRIN^210^ may retain the binding affinity due to other residues or regions within the protein that are able to compensate for the loss of the interacting site. This compensatory mechanism is sometimes seen in other protein interactions, where other binding sites or alternative residues can contribute to the interaction when the primary binding site is not available [59]. This can be further explained by the notable decrease in interaction between D180A and LTβR, indicating that the involvement of the second EF-hand motif in ΔLTRIN in binding to LTβR plays a role. However, the fact that the double mutant D123A D180A restored the interaction to wild-type levels suggests that the D123A mutation may enhance the interaction with LTβR. Overall, these findings highlight the complex nature of the interaction between ΔLTRIN and LTβR, involving multiple residues and regions within ΔLTRIN that contribute to binding affinity. Secondly, similar to what was observed with FL LTRIN, the shorter random coil N-terminal of ΔLTRIN could still affect the interaction as well. In particular, removing residues from 91 to 118 exposes more of the hindered interacting site and facilitates its binding with LTβR [60].

## 5. Conclusions

**Figure 7:**
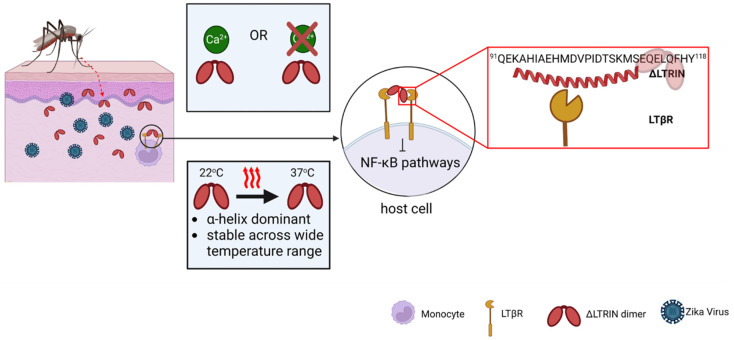
ΔLTRIN is injected together with ZIKV during a blood meal of the infected mosquito. ΔLTRIN exists as a homodimer and its secondary structure remains unchanged regardless of the presence of Ca^2+^. The secondary structure exhibits resilience to varying temperatures, allowing ΔLTRIN to maintain its full functionality from mosquito (22 °C) to human host (37 °C). The interacting sequence of ΔLTRIN is identified by HDX-MS with sequence ^91^QEKAHIAEHMDVPIDTSKMSEQELQFHY^118^ from the N-terminal. Illustration created with BioRender.com.

In summary, we demonstrate that ΔLTRIN exists as a homodimer. Despite being an EF-hand protein, its secondary structure is not affected by binding to Ca^2+^. Through ELISA assay and HDX-MS, we identified the binding region of ΔLTRIN with LTβR. Although the presence of Ca^2+^ does not affect the binding affinity, disrupting the calcium-binding ability specifically in the second EF-hand motif significantly affects the interaction between ΔLTRIN and LTβR (Figure 7). The decrease in binding affinity of FL LTRIN explains the reason behind the presence of only the truncated form, ΔLTRIN, in the saliva of mosquitoes as the N-terminal region hinders its interaction. Our findings provide mechanistic insight into the interactions between LTRIN and LTβR, which will aid in the development of inhibitors against ΔLTRIN as a potential treatment or vaccine target for ZIKV. Understanding the binding mechanism of LTRIN with LTβR sheds light on how specific regions play a role in mediating this interaction.

## Data Availability

Data are contained within the article and Supplementary Materials.

## Supplementary Materials

The following supporting information can be downloaded at: https://www.mdpi.com/article/10.3390/biology13010042/s1, Figure S1: (a) Sedimentation equilibrium analysis of ΔLTRIN indicates that it exists in dimer formation. (b) Dynamic light scattering data (DLS) of ΔLTRIN across different concentration. (c) Size exclusion chromatography elution profile and SDS-PAGE of ΔLTRIN in the presence of Ca^2+^ or EGTA. (d) Comparing the DLS data of ΔLTRIN in the presence of Ca^2+^ or EGTA. (e) Size exclusion chromatography elution profile and SDS-PAGE C133Q ΔLTRIN. (f) DLS data of C133Q ΔLTRIN showing that it is in dimer form. (g) The CD spectra of ΔLTRIN in the presence of 10 mM EGTA continue to exhibit a predominant alpha helix secondary structure. (h) Secondary structure prediction of full length LTRIN by PSIPRED. The signal peptide is highlighted in red box. The ΔLTRIN used is underlined with green; Figure S2: The EF-hand motif of ΔLTRIN (a) Ca^2+^ binding ability of ΔLTRIN is visualised using ruthenium red stanning. (b) Gel mobility shift assay of ΔLTRIN shows that limited structural changes are induced upon bind with Ca^2+^. (c) ΔLTRIN shows different binding affinity towards different divalent ions. (d) The presence of Zn^2+^ causes protein precipitation, resulting in a negative outcome; Table S1: The CD spectra of ΔLTRIN in the presence of 2 mM CaCl_2_ is employed to assess its secondary structure content through three different methods (SELCON3, CONTINLL, and CDSSTR). The proportion of alpha-helix and loop structures in ΔLTRIN as anticipated by AlphaFold was also quantified.
