# Supplementary material for: Recognition of Aedes aegypti Mosquito Saliva Protein LTRIN by the Human Receptor LTβR for Controlling the Immune Response
Source: Biology (Basel). 2024 Jan 12;13(1):42. doi: 10.3390/biology13010042 (PMC10813304; doi:10.3390/biology13010042)
Supplement: Supplementary file 1 [file biology-13-00042-s001.zip › biology-2779722-supplementary.pdf]

Supplementary Figure

a

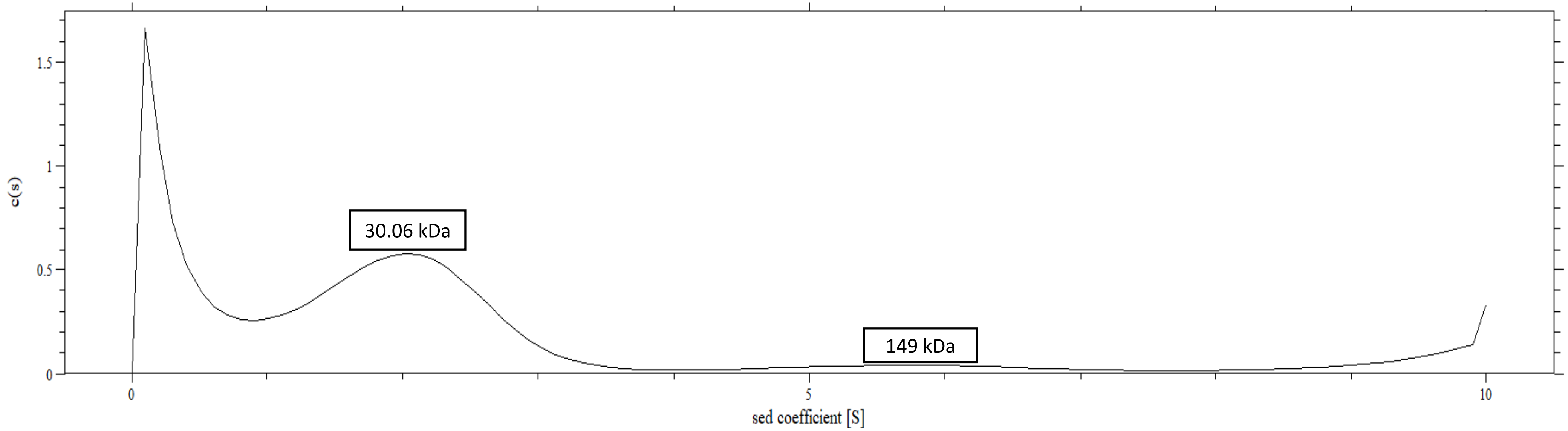

Supplementary Figure S1: a) Sedimentation equilibrium analysis of ΔLTRIN indicates that it exist in dimer formation. b) Dynamic light scattering data (DLS) of ΔLTRIN across different concentration. c) Size exclusion chromatography elution profile and SDS-PAGE of ΔLTRIN in the presence of Ca^{2+} or EGTA. d) Comparing the DLS data of ΔLTRIN in the presence of Ca^{2+} or EGTA. e) Size exclusion chromatography elution profile and SDS-PAGE C133Q ΔLTRIN. f) DLS data of C133Q ΔLTRIN showing that it is in dimer form. g) The CD spectra of ΔLTRIN in the presence of 10mM EGTA continue to exhibit a predominant alpha helix secondary structure. h) Secondary structure prediction of full length LTRIN by PSIPRED. The signal peptide is highlighted in red box. The ΔLTRIN used is underlined with green.

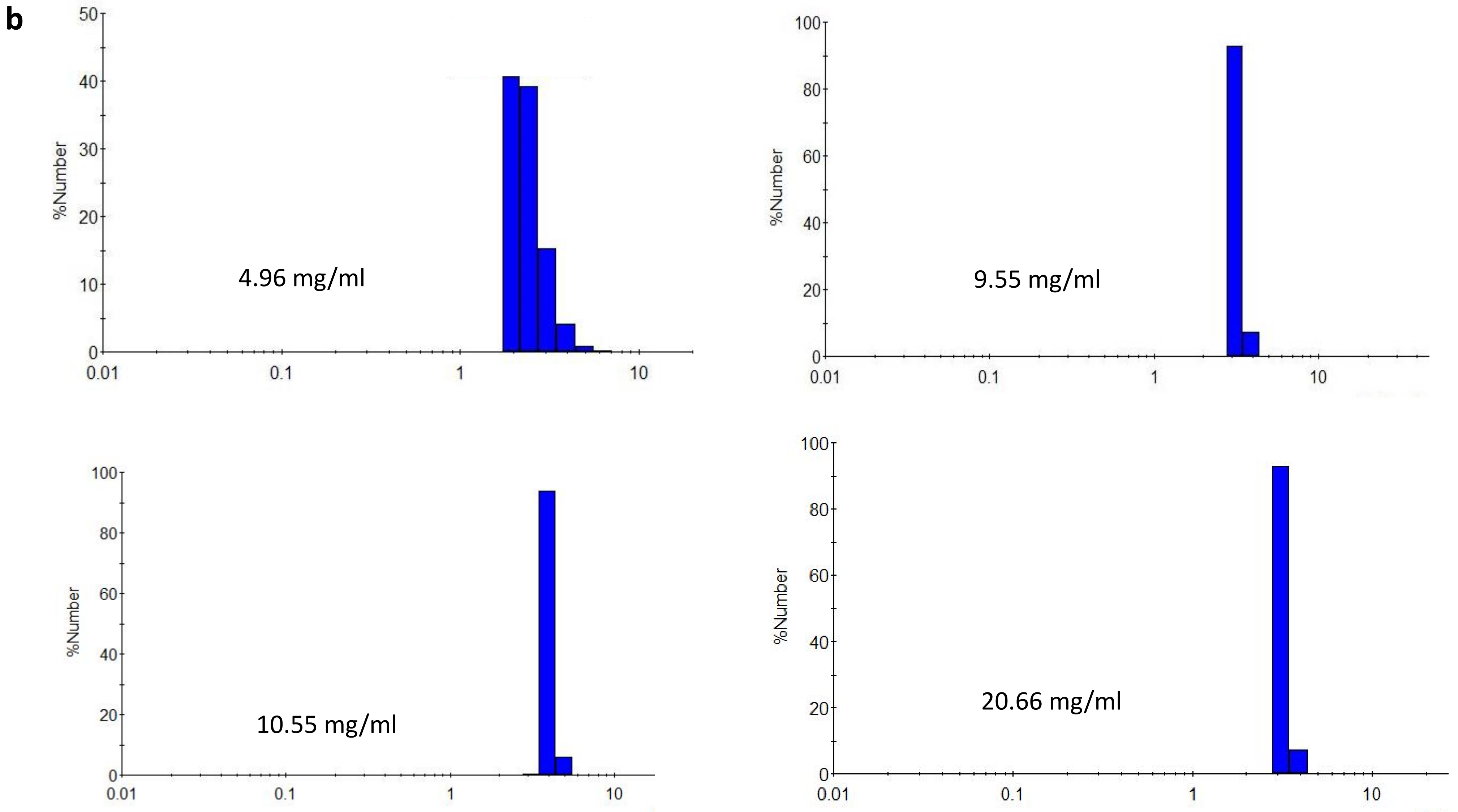

Supplementary Figure S1: a) Sedimentation equilibrium analysis of ΔLTRIN indicates that it exist in dimer formation. b) Dynamic light scattering data (DLS) of ΔLTRIN across different concentration. c) Size exclusion chromatography elution profile and SDS-PAGE of ΔLTRIN in the presence of Ca^{2+} or EGTA. d) Comparing the DLS data of ΔLTRIN in the presence of Ca^{2+} or EGTA. e) Size exclusion chromatography elution profile and SDS-PAGE C133Q ΔLTRIN. f) DLS data of C133Q ΔLTRIN showing that it is in dimer form. g) The CD spectra of ΔLTRIN in the presence of 10mM EGTA continue to exhibit a predominant alpha helix secondary structure. h) Secondary structure prediction of full length LTRIN by PSIPRED. The signal peptide is highlighted in red box. The ΔLTRIN used is underlined with green.

c

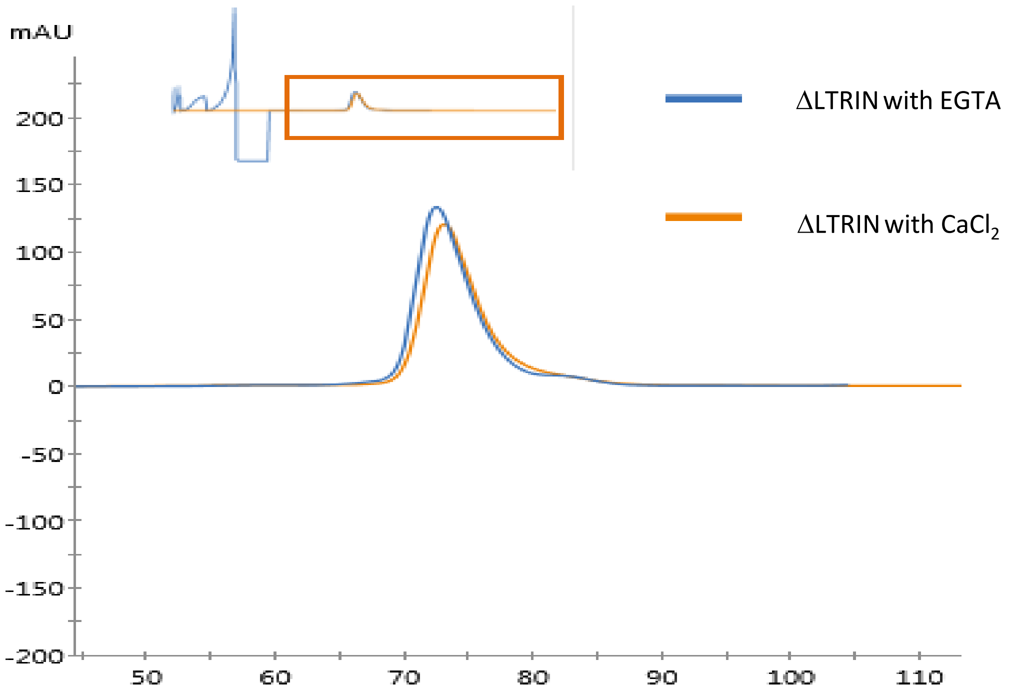

Supplementary Figure S1: a) Sedimentation equilibrium analysis of ΔLTRIN indicates that it exist in dimer formation. b) Dynamic light scattering data (DLS) of ΔLTRIN across different concentration. c) Size exclusion chromatography elution profile and SDS-PAGE of ΔLTRIN in the presence of Ca^{2+} or EGTA. d) Comparing the DLS data of ΔLTRIN in the presence of Ca^{2+} or EGTA. e) Size exclusion chromatography elution profile and SDS-PAGE C133Q ΔLTRIN. f) DLS data of C133Q ΔLTRIN showing that it is in dimer form. g) The CD spectra of ΔLTRIN in the presence of 10mM EGTA continue to exhibit a predominant alpha helix secondary structure. h) Secondary structure prediction of full length LTRIN by PSIPRED. The signal peptide is highlighted in red box. The ΔLTRIN used is underlined with green.

d

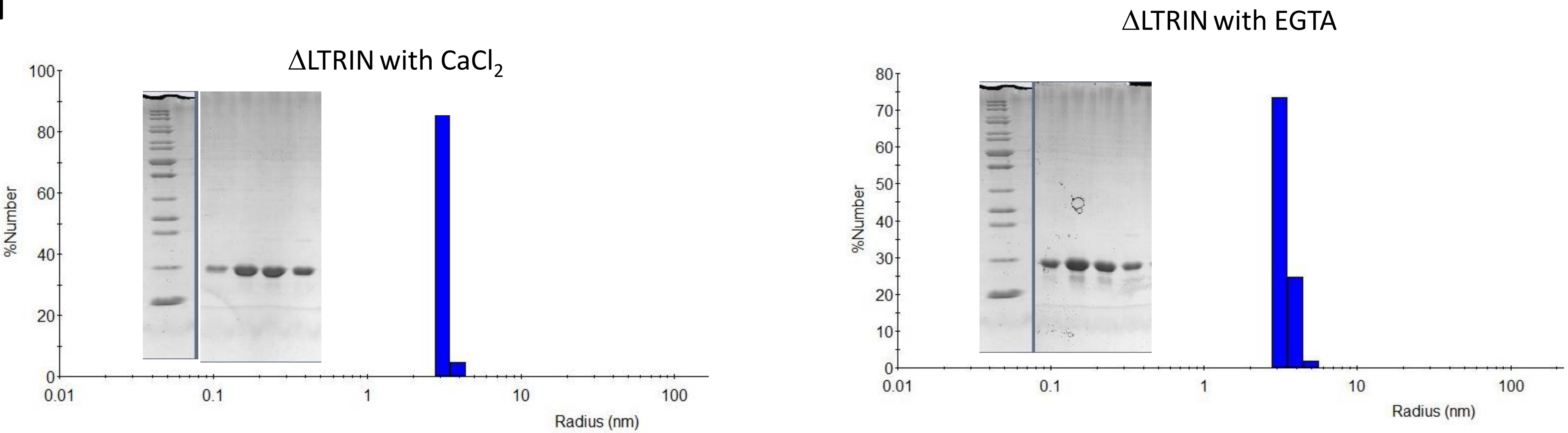

Supplementary Figure S1: a) Sedimentation equilibrium analysis of ΔLTRIN indicates that it exist in dimer formation. b) Dynamic light scattering data (DLS) of ΔLTRIN across different concentration. c) Size exclusion chromatography elution profile and SDS-PAGE of ΔLTRIN in the presence of Ca^{2+} or EGTA. d) Comparing the DLS data of ΔLTRIN in the presence of Ca^{2+} or EGTA. e) Size exclusion chromatography elution profile and SDS-PAGE C133Q ΔLTRIN. f) DLS data of C133Q ΔLTRIN showing that it is in dimer form. g) The CD spectra of ΔLTRIN in the presence of 10mM EGTA continue to exhibit a predominant alpha helix secondary structure. h) Secondary structure prediction of full length LTRIN by PSIPRED. The signal peptide is highlighted in red box. The ΔLTRIN used is underlined with green.

e

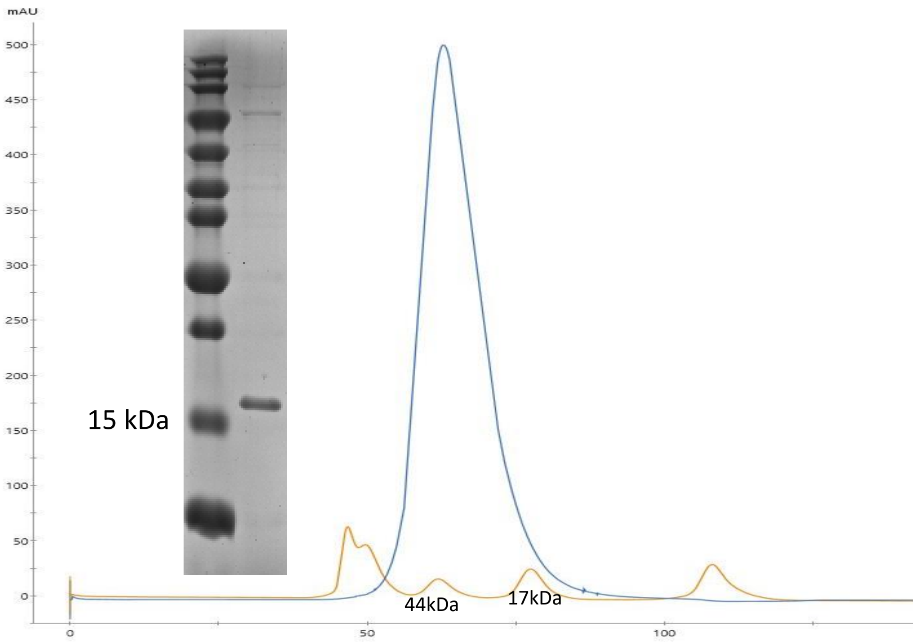

Supplementary Figure S1: a) Sedimentation equilibrium analysis of ΔLTRIN indicates that it exist in dimer formation. b) Dynamic light scattering data (DLS) of ΔLTRIN across different concentration. c) Size exclusion chromatography elution profile and SDS-PAGE of ΔLTRIN in the presence of Ca^{2+} or EGTA. d) Comparing the DLS data of ΔLTRIN in the presence of Ca^{2+} or EGTA. e) Size exclusion chromatography elution profile and SDS-PAGE C133Q ΔLTRIN. f) DLS data of C133Q ΔLTRIN showing that it is in dimer form. g) The CD spectra of ΔLTRIN in the presence of 10mM EGTA continue to exhibit a predominant alpha helix secondary structure. h) Secondary structure prediction of full length LTRIN by PSIPRED. The signal peptide is highlighted in red box. The ΔLTRIN used is underlined with green.

f

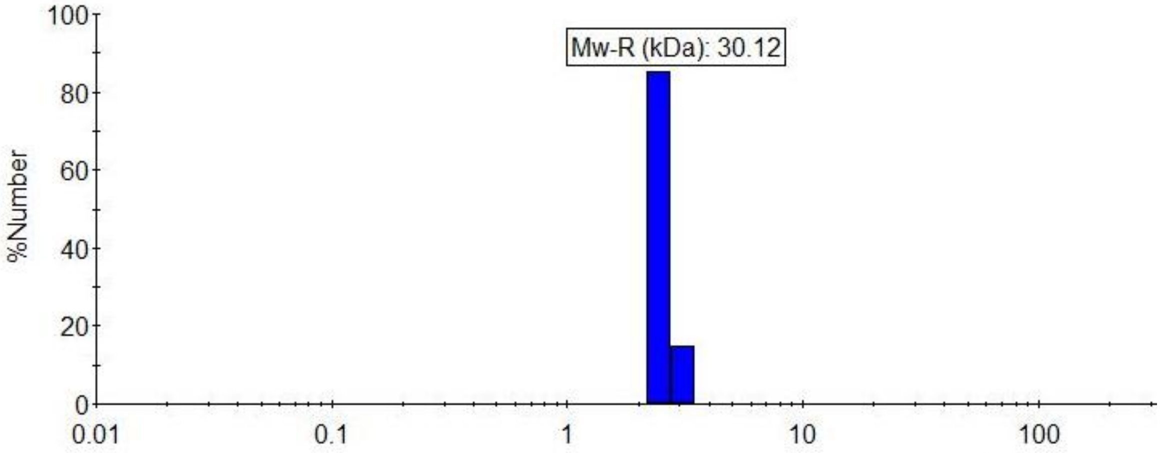

Supplementary Figure S1: a) Sedimentation equilibrium analysis of ΔLTRIN indicates that it exist in dimer formation. b) Dynamic light scattering data (DLS) of ΔLTRIN across different concentration. c) Size exclusion chromatography elution profile and SDS-PAGE of ΔLTRIN in the presence of Ca^{2+} or EGTA. d) Comparing the DLS data of ΔLTRIN in the presence of Ca^{2+} or EGTA. e) Size exclusion chromatography elution profile and SDS-PAGE C133Q ΔLTRIN. f) DLS data of C133Q ΔLTRIN showing that it is in dimer form. g) The CD spectra of ΔLTRIN in the presence of 10mM EGTA continue to exhibit a predominant alpha helix secondary structure. h) Secondary structure prediction of full length LTRIN by PSIPRED. The signal peptide is highlighted in red box. The ΔLTRIN used is underlined with green.

g

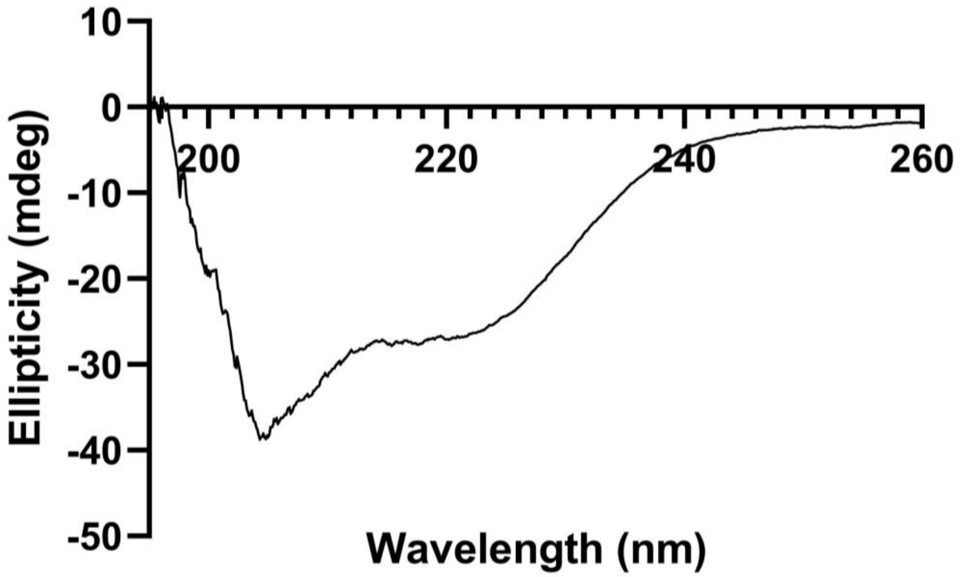

Supplementary Figure S1: a) Sedimentation equilibrium analysis of ΔLTRIN indicates that it exist in dimer formation. b) Dynamic light scattering data (DLS) of ΔLTRIN across different concentration. c) Size exclusion chromatography elution profile and SDS-PAGE of ΔLTRIN in the presence of Ca^{2+} or EGTA. d) Comparing the DLS data of ΔLTRIN in the presence of Ca^{2+} or EGTA. e) Size exclusion chromatography elution profile and SDS-PAGE C133Q ΔLTRIN. f) DLS data of C133Q ΔLTRIN showing that it is in dimer form. g) The CD spectra of ΔLTRIN in the presence of 10mM EGTA continue to exhibit a predominant alpha helix secondary structure. h) Secondary structure prediction of full length LTRIN by PSIPRED. The signal peptide is highlighted in red box. The ΔLTRIN used is underlined with green.

h

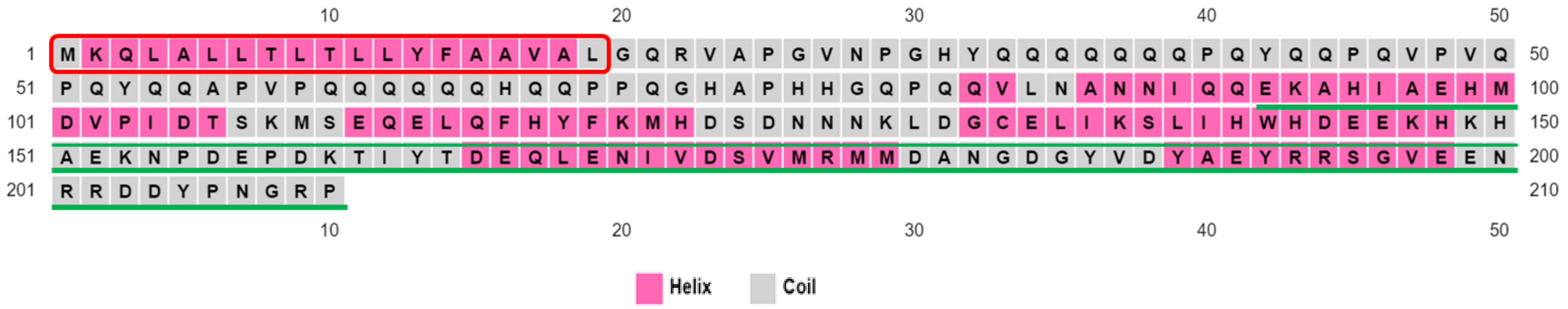

Supplementary Figure S1: a) Sedimentation equilibrium analysis of ΔLTRIN indicates that it exist in dimer formation. b) Dynamic light scattering data (DLS) of ΔLTRIN across different concentration. c) Size exclusion chromatography elution profile and SDS-PAGE of ΔLTRIN in the presence of Ca^{2+} or EGTA. d) Comparing the DLS data of ΔLTRIN in the presence of Ca^{2+} or EGTA. e) Size exclusion chromatography elution profile and SDS-PAGE C133Q ΔLTRIN. f) DLS data of C133Q ΔLTRIN showing that it is in dimer form. g) The CD spectra of ΔLTRIN in the presence of 10mM EGTA continue to exhibit a predominant alpha helix secondary structure. h) Secondary structure prediction of full length LTRIN by PSIPRED. The signal peptide is highlighted in red box. The ΔLTRIN used is underlined with green.

a

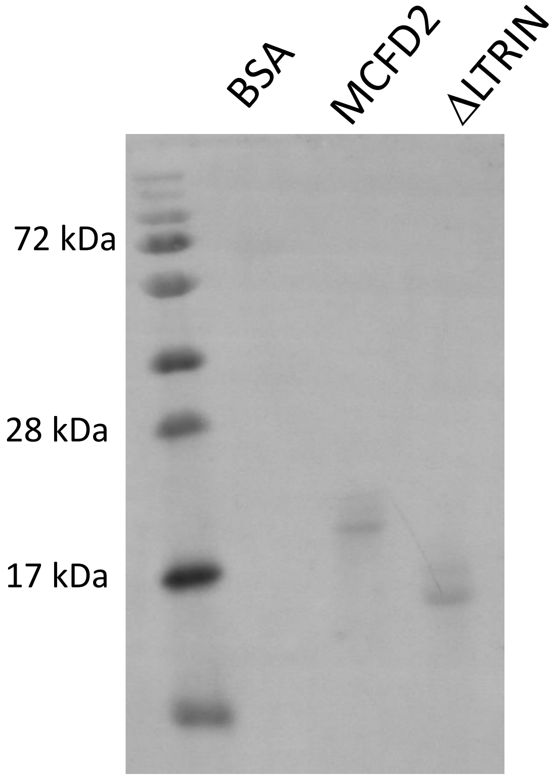

Supplementary Figure S2: The EF-hand motif of ΔLTRIN a) Ca^{2+} binding ability of ΔLTRIN is visualised using ruthenium red stanning. b) Gel mobility shift assay of ΔLTRIN shows that limited structural changes is induced upon bind with Ca^{2+}. c) ΔLTRIN shows different binding affinity towards different divalent ions. d) The presence of Zn2+ causes protein precipitation, resulting in a negative outcome.

b

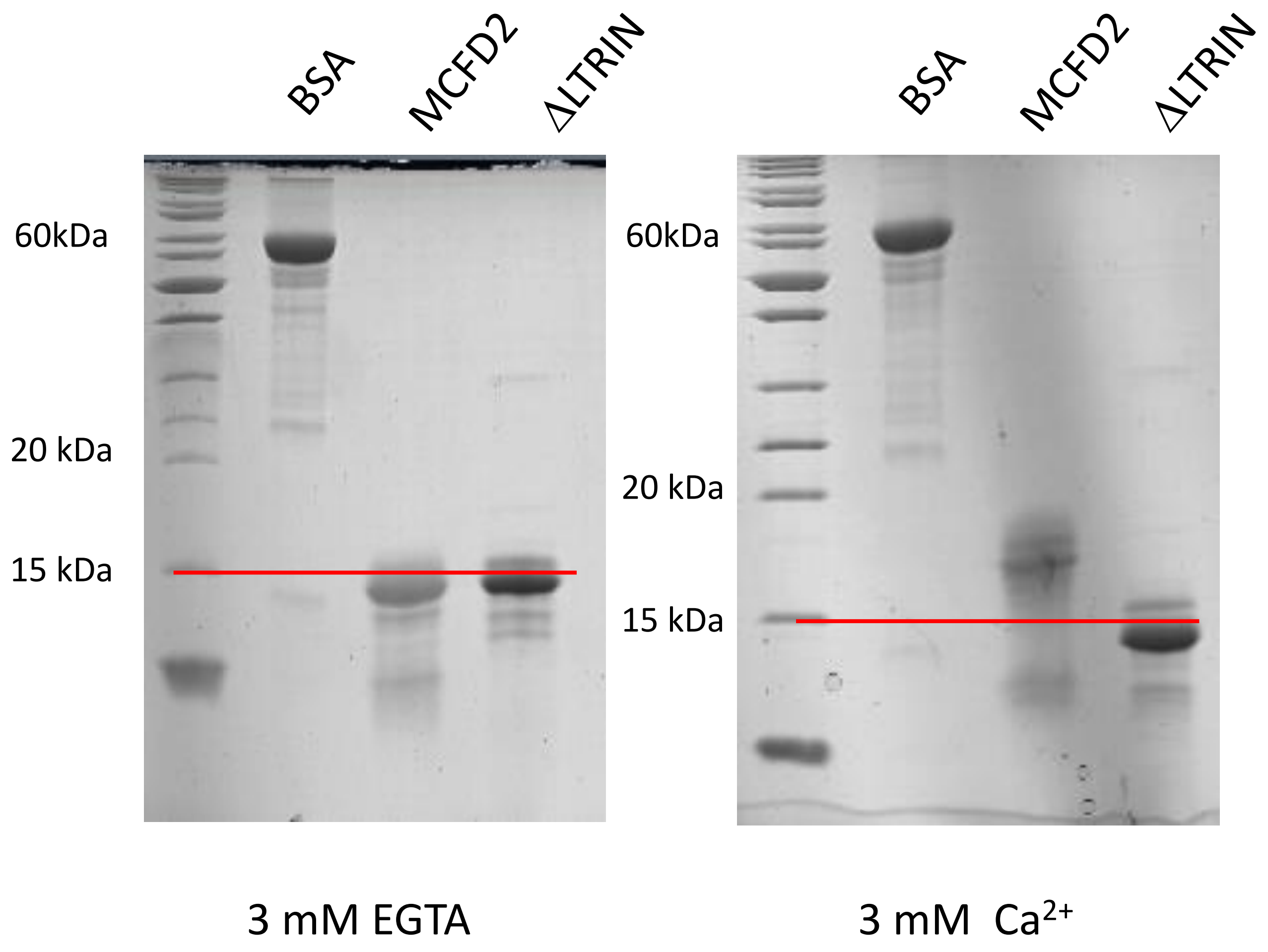

Supplementary Figure S2: The EF-hand motif of ΔLTRIN a) Ca^{2+} binding ability of ΔLTRIN is visualised using ruthenium red stanning. b) Gel mobility shift assay of ΔLTRIN shows that limited structural changes is induced upon bind with Ca^{2+}. c) ΔLTRIN shows different binding affinity towards different divalent ions. d) The presence of Zn2+ causes protein precipitation, resulting in a negative outcome.

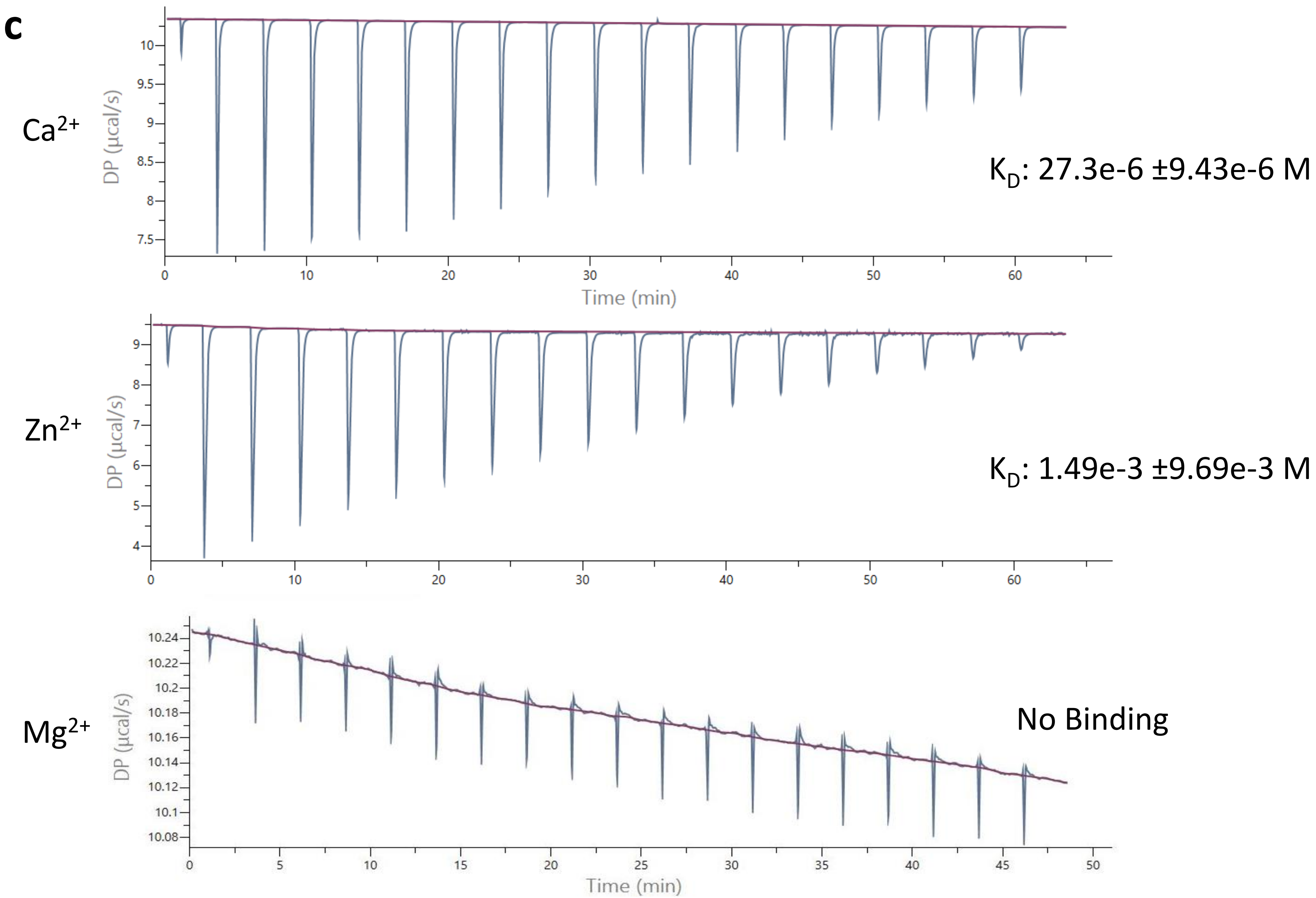

Supplementary Figure S2: The EF-hand motif of ΔLTRIN a) Ca^{2+} binding ability of ΔLTRIN is visualised using ruthenium red stanning. b) Gel mobility shift assay of ΔLTRIN shows that limited structural changes is induced upon bind with Ca^{2+}. c) ΔLTRIN shows different binding affinity towards different divalent ions. d) The presence of Zn2+ causes protein precipitation, resulting in a negative outcome.

d

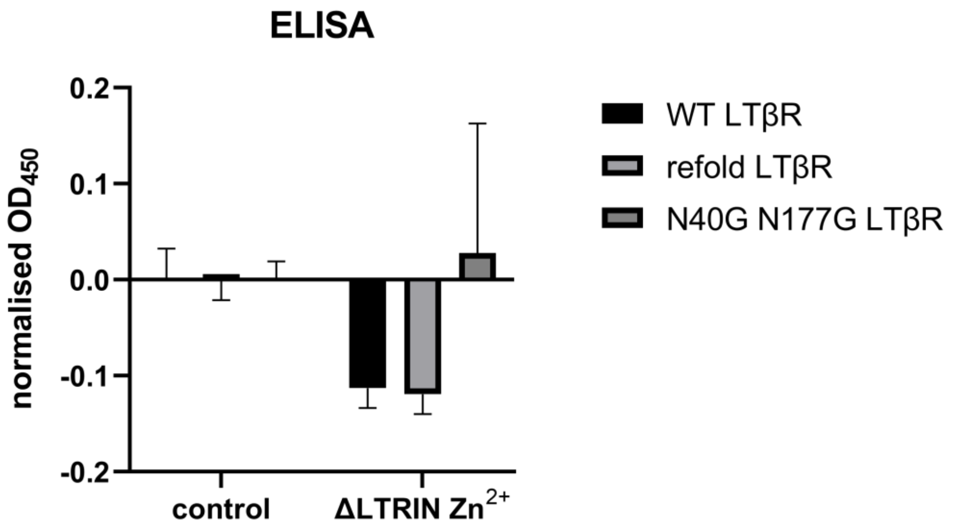

Supplementary Figure S2: The EF-hand motif of ΔLTRIN a) Ca^{2+} binding ability of ΔLTRIN is visualised using ruthenium red stanning. b) Gel mobility shift assay of ΔLTRIN shows that limited structural changes is induced upon bind with Ca^{2+}. c) ΔLTRIN shows different binding affinity towards different divalent ions. d) The presence of Zn^{2+} causes protein precipitation, resulting in a negative outcome.

	H(r)	H(d)	S(r)	S(d)	Turn	Unrd
SELCON3	0.214	0.143	0.044	0.070	0.245	0.323
CONTINLL	0.264	0.462	0.00	0.053	0.221	0.00
CDSSTR	0.300	0.257	0.059	0.094	0.151	0.145
AlphaFold	0.586		0.00		0.414	

Supplementary Table S1: The CD spectra of ΔLTRIN in the presence of 2mM CaCl2 is employed to assess its secondary structure content through three different methods (SELCON3, CONTINLL, and CDSSTR). The proportion of alpha-helix and loop structures in ΔLTRIN as anticipated by AlphaFold was also quantified. H(r): regular alpha helices; H(d): distorted alpha helices; S(r): regular beta-sheets; S(d): distorted beta-sheets; Turn: beta-turn; Unrd: unordered structure.
